# Elimination of violence against women and girls as a global action agenda

**DOI:** 10.5249/jivr.v9i2.908

**Published:** 2017-07

**Authors:** Yadlapalli S. Kusuma, Bontha V. Babu

**Affiliations:** ^*a*^Centre for Community Medicine, All India Institute of Medical Sciences, New Delhi, India.; ^*b*^Socio-Behavioural & Health Systems Research Division, Indian Council of Medical Research, New Delhi, India.

**Keywords:** Preparedness, Sustainable development goals, Gender, Violence, Women’s health

## Abstract

This article outlines the goals and targets of Sustainable Development Goals (SDGs) related to elimination of violence against women and girls (VAWG) and to explain the framework to target these goals. Prevention of VAWG has been identified as one of the key agents for sustainable development. SDGs gave enough importance and called for the elimination of “all forms of violence against all women and girls everywhere”. It identified different social and political enablers of reducing violence which are targeted under different SDGs. This acknowledges tacitly that VAWG is preventable and it is set to prevent and eliminate it. Evidences show that preventing VAWG is possible through multi-sectorial programs. The United Nations committed to revitalized global partnership to mobilize resources for implementing the agenda. Hence, designing and implementing interventions and subsequently scaling-up and intensifying these interventions are required to end VAWG.

## Introduction

The 2030 Agenda for Sustainable Development was adopted by the 193 member states of the United Nations (UN) on 25 September 2015. The Sustainable Development Goals (SDGs), a set of 17 goals and 169 targets provide the nations with a framework for advancing sustainable development in three dimensions –economic, social and environmental – over the next 15 years.^1^ The SDGs replace the Millennium Development Goals (MDGs), which were operational for 15 years. The MDGs provided frameworks for development and considerable progress has been made in number of areas. However, this progress has been uneven in several less developed countries and some goals remain off-tracked. ^2^ In SDGs, the prevention of violence against women and girls (VAWG) took an important place, compared to MDGs. MDGs provide only a framework within which the impact of violence could be measured against other developmental dimensions. MDG-3 specified to promote gender equality and empower women with a target to eliminate gender disparity in education; however, the other goals too have implications to prevent gender discrimination. Though the indicators of gender equality have also been improved after implementing MDGs, gender disparities still exist. ^2^ The aim of the present paper is to outline goals and targets of SDGs related to the elimination of VAWG and to explain the framework to target these goals through achieving gender equality and empowering women and girls. This paper also explains briefly the global efforts against gender inequalities. 

The SDGs have the potential to achieve more than MDGs in terms of their scope, aspiration and vision. The SDG-5, the ‘gender goal’ exclusively deals with achieving gender equality and empowering women and girls. It is targeted to eliminate all forms of VAWG including trafficking and sexual and other forms of exploitation (target 5.2), and harmful practices such as child, early and forced marriages and female genital mutilation (target 5.3). Though, it is the legacy of MDG-3, this SDG is formulated on strong gender framework with an understanding of gender inequality to possess economic, political and social aspects, which are interlinked. The strong wording of SDG-5 (‘achieve gender equality’) than of MDG-3 (‘promote gender equality’) reveals its commitment. The SDG-16, which is meant to promote peaceful and inclusive societies for sustainable development, included a goal to end abuse, exploitation, trafficking and all forms of violence against and torture of children (target 16.2). This goal is tackled with another omission of MDGs – gender in governance, participation, inclusion, rights and security. And this goal mainly concerned on female children, who are at risk of all forms of violence. The target 16.1 is meant to reduce all forms of violence and related deaths to attain a peaceful and inclusive societies. The SDG-16 addresses reducing violence and related deaths among women including deaths related to domestic violence and dowry, which are rampant in many developing countries.^3^ Thus, these 4 SDG targets (5.2, 5.3, 16.1 and 16.2) directly targeted VAWG, and there are several targets among the other SDGs that were directly or indirectly aimed to prevent and reduce VAWG. These targets are to ensure that all men and women have equal rights to resources, basic services, ownership and control over properties, inheritance, natural resources, technology, etc. (target 1.4); create policy frameworks based on gender-sensitive developmental strategies (target 1b), double agricultural productivity/income in small-scale food producers, particularly women through secure and equal access to land and other resources (target 2.3); ensure universal access to sexual and reproductive healthcare, and related services and rights (targets 3.7 and 5.6); ensure that all girls and boys complete free, equitable and quality primary and secondary education (target 4.1), childhood development, care and pre-primary education (target 4.2), technical, vocational and tertiary/university education (target 4.3), and to eliminate gender disparity in education (target 4.5); target all forms of discrimination against women and girls everywhere (target 5.1); recognize and value unpaid care and domestic work (carried out by women) (target 5.4); ensure women’s participation/leadership in decision making in all spheres (target 5.5); undertake reforms to give women equal rights and access to economic resources (target 5a); enhance technology to empower women (target 5b); adopt and strengthen policies and enforceable legislation towards gender equality and women empowerment (target 5c); empower and promote the social, economic and political inclusion of all, irrespective of age, sex, disability, race, ethnicity, origin, religion or economic or other status (target 10.2); ensure equal opportunity and reduce inequalities of outcome, including by eliminating discriminatory laws, policies and practices and promoting appropriate legislation, policies and action in this regard (target 10.3); provide universal access to safe public transport (target 11.2) and public spaces, in particular for women (target 11.7). ^1^

Thus, the gender equality has been identified as one of the key agents for sustainable development. Many SDGs and their targets are related to gender as no economic, social, political or ecological issue is gender neutral. Hence, SDGs gave enough importance to prevention of VAWG. This developmental framework identified different economic, social, political and ecological enablers of reducing VAWG and is targeted under different SDGs. Thus, it recognized the impact of VAWG on well-being of women in specific and on development agenda, in general,^4^ and acknowledged tacitly that this gender-based violence is preventable. This is an important recognition to the campaigns against VAWG and efforts of social researchers and activists. More than 30% of women worldwide have experienced either or both physical and sexual violence. ^5^ And 38.6% of all female murders worldwide are estimated to be perpetuated by intimate partners. ^6^ Around 20% of women report being sexually abused as children and 7% of women worldwide are thought to have been sexually assaulted by some or other than partner since 15 years of age. ^7,8^ An estimated 11.4 million women and girls are trafficked worldwide. ^9^ About 70 million girls worldwide have been married before the age of 18 years, mostly against their will. ^10^ In addition, several deaths of women and girls cited related to honor killing in Pakistan and North Indian states. ^11,12^ Up to 140 million girls and women undergone genital mutilation and more than 3 million girls are at risk of genital mutilation every year in Africa alone. ^13^ All these are consequent of gender inequalities that are deep rooted in almost all societies, and women mostly denied access to several services including health care. Women who are exposed to violence need higher utilization of health care services. ^14,15^ VAWG is a barrier to women’s equal participation in society and affects overall development. ^16^ The short- and long-term impacts of exposure to VAWG are multiple. It is leading cause of homicides in women. ^6^ It is increased cause of several mental health problems including depression and suicidal behaviour. ^5,17^ The women and girls exposed to violence are more likely to acquire HIV. ^18,19^ In addition, the VAWG has lot of economic impact. ^20^ The estimates of lost productivity from VAWG are - 1.2% of the gross domestic product in Brazil and Tanzania, 2% in Chile ^21,22^ and 0.9-1.3% in South Africa. ^23^ The annual costs related to health care, legal, police and social services to address VAWG was estimated to be $ 5.8 billion in the USA in 2003, UK£ 22.9 billion in England and Wales in 2004; UK£ 4.5 billion in Australia in 2004. ^24,25^

It is to be noted that it is not the beginning of the war against gender inequalities. The UN, since its creation about 70 years ago, has made attempts towards advancing gender equality from the institution of the Commission on the Status of Women (an intergovernmental body dedicated to the promotion of gender equality and the empowerment of women) through adoption of various landmark agreements like Convention on the Elimination of All Forms of Discrimination Against Women (CEDAW) and the Beijing Declaration and Platform for Action.^26^ Another global attempt is the 67th World Health Assembly (WHA)'s resolution to strengthen the role of the health systems in addressing violence specifically against women and girls.^27^ Now, through SDGs, it is set to prevent and eliminate VAWG, however along with several complex targets. This new agenda requires a revitalized global partnership and the UN General Assembly committed to it for mobilizing the means required to implement the agenda. This partnership will ensure the solidarity with the poorest and the most vulnerable countries and populations. This partnership will further facilitate an intensive global engagement bringing together governments, the private sector, civil society, the UN and other developmental actors, and mobilizing resources.^28^ Though the agenda recognized that each country has primary responsibility for its development, it ensured the global support particularly to the poorest and most vulnerable countries in mobilizing resources and enhancing their participation. ^28^

Thus, the advantage of inclusion of VAWG in SDGs would be increased intensity of campaigns and more funds and resources for their campaigns and research. Countries are developing action plans to address VAWG along with other SDG targets by involving various developmental partners. The agenda of actions against VAWG will reflect in countries’ policy documents and thereby explicit interventions. Evidences based on existing interventions and programs show that preventing VAWG is possible. Interventions should adopt a holistic and comprehensive approach to address the drivers and consequents of VAWG, empower women and reduce their vulnerability. In addition, efforts are needed to promote coordination between all relevant actors including health, education, police, judiciary and community groups. This kind of multi-sectorial approach is recognized as effective in addressing VAWG. ^29^ Multi-sectorial programs to promote gender equality and women empowerment seem to be successful to transform deeply entrenched attitudes and behaviours. ^30^ However, action plans of such programs need to be guided by the principles of gender equality, human rights and health equity. ^31^ Such programs not only challenge the acceptability of violence, but also address underlying risk factors. ^30^ Health sector can support programs that involve women and children affected by violence. Health care system can provide women with a safe-environment where they can confidentially disclose their violence experience and seek a supportive response. ^31,32^ It is evident that some health sector based interventions can have positive outcomes for women such as reduction in victimization by intimate partner, ^33,34^ reduction of revictimization ^35,36^ and reduction in mental health problems such as depression, ^34,35,37,38^ etc. In 2013, WHO issued clinical and policy guidelines for responding to VAWG. ^39^ These guidelines are on identification of survivors of violence and clinical care for survivors of different forms of violence. These guidelines can be used as basis for developing or updating their national guidelines and protocols for health sector response to VAWG. These guidelines need to be accompanied by provision of accessible, affordable, quality and comprehensive health care services for women and girls, including those living in humanitarian settings. ^31^ Health sector should also address other behavioral risk factors such as alcohol and substance abuse. ^40^ The mental health program should be strengthened to support victims of VAWG. ^41,42,43^ Thus, several opportunities and plans are available to gear up the actions against VAWG. ^44^ These efforts and plans are to be consolidated during developing implementation plans at different levels in countries. However, the policy directions under developmental framework should be available from global partners. Subsequently, the national and sub-national efforts are to be scaled-up and intensified to achieve the targets towards eliminating the VAWG.
